# Association of a Modified Blumgart Anastomosis With the Incidence of Pancreatic Fistula and Operation Time After Laparoscopic Pancreatoduodenectomy: A Cohort Study

**DOI:** 10.3389/fsurg.2022.931109

**Published:** 2022-06-27

**Authors:** Yong-Gang He, Xiao-Min Yang, Xue-Hui Peng, Jing Li, Wen Huang, Gui-Cang Jian, Jing Wu, Yi-Chen Tang, Liang Wang, Xiao-Bing Huang

**Affiliations:** Department of Hepatobiliary, The Second Affiliated Hospital of Army Medical University, Chongqing, China

**Keywords:** blumgart anastomosis, laparoscopic pancreatoduodenectomy, pancreaticojejunostomy, POPF, operation time

## Abstract

**Objective:**

To explore the association between a modified Blumgart anastomosis technique and the operative time and surgical complications.

**Methods:**

This is a retrospective cohort study that analyzed the data of patients who underwent laparoscopic pancreaticoduodenectomy from January 2015 to March 2021. The primary outcome was to explore the association between the modified Blumgart anastomosis technique and operative time.

**Results:**

A total of 282 patients were enrolled. There were 177 cases of pancreatic duct-to-mucosa anastomosis in the traditional surgery group, and 105 cases of the modified three-step Blumgart anastomosis in the modified group. There were no statistically significant differences in the general and intraoperative characteristics found between the two groups (*P* > 0.05). The surgical method was an independent predictor of operative time. Overall complications postsurgery were less common in the modified group than in the traditional group. The incidence of postoperative pancreatic fistula was higher in the traditional group than in the modified group (45 cases (25.4%) and 11 cases (10.5%), respectively). Fourteen cases (7.9%) in the traditional group and four case (3.8%) in the modified group had postoperative pancreatic fistula of grades B + C. The two groups had statistically significant differences (*P* < 0.05). The results of the linear regression showed that the type of surgical method was associated with operation time (95% CI, −73.074 to −23.941, β: −0.438, *P* < 0.001).

**Conclusion:**

This modified three-step Blumgart pancreaticojejunostomy was associated with the operation time.

## Introduction

Laparoscopic pancreaticoduodenectomy has undergone gradual developments in recent years, owing to improvements in surgical instruments, the surgical skills and expertise of surgeons, and perioperative management. Pancreaticoduodenectomy (PD) is a challenging but standard surgical method that is used to treat benign and malignant diseases at the pancreatic head and ampulla (1, 2). However, the postoperative complications and mortality rate associated with PD are high due to its wide range of surgical resections, complicated processes, and considerable trauma (1, 2). With improvements in surgical techniques and perioperative management, the mortality rate of PD has decreased. However, the incidence of postoperative complications remains as high as 30%–50% (2, 3), especially that of postoperative pancreatic fistula(POPF), which is at 5%–40% (3). POPF is one of the most serious complications of PD. It is characterized by a leakage of pancreatic fluid as a result of damaged pancreatic ducts after surgery. It can cause life-threatening complications, such as ascites and pancreatic fluid-induced corrosion of peripheral blood vessels, surgical wounds and related abdominal bleeding, abdominal infection, and abdominal abscess. Treating these conditions can result in a prolonged hospitalization, increased medical expenses, and even hospital death in serious cases (4, 5).

Several studies have shown that compared to open PD, laparoscopic PD has certain advantages, such as less trauma, quick recovery, less bleeding, and a good postoperative quality of life. With the development of modern surgery techniques and the advanced skills of experienced pancreatic surgeons, PD has become a safe and feasible procedure (6). Laparoscopic PD involves the same pancreaticojejunostomy procedure that used in open PD. At present, there are many surgical techniques that can reduce the incidence of POPF by improving the pancreaticojejunostomy technique. The commonly used surgical methods of pancreaticojejunostomy include invaginated pancreaticojejunostomy (7) and pancreatic duct-to-jejunal mucosa anastomosis (8). Since the development of laparoscopic PD, Wang (9) and Cai (10) have reported an improved technique of laparoscopic pancreaticojejunostomy with fewer complications. We aimed to explore and investigate the association between pancreatic duct-to-mucosa anastomosis in the traditional surgery group and the modified three-step Blumgart anastomosis modified group and the operative time and surgical complications.

## Methods

### Study Design and Patients

This was a retrospective cohort study. Patients diagnosed with pancreatic tumors, ampulla of Vater tumors, bile duct tumors, and pancreatic duct stones were analyzed by imaging examination at the Department of Hepatobiliary Surgery of the Second Affiliated Hospital of Army Medical University from January 2015 to March 2021.

The inclusion criteria were as follows: (1) pancreatic head or periampullary tumor clearly diagnosed without signs of distant metastasis, based on preoperative imaging; (2) all patients underwent laparoscopic PD and possessed results of their pathological examination; (3) the tumor did not invade peripheral blood vessels, such as the portal vein and the superior mesenteric artery and vein, and did not require combined vascular and/or organ resection; (4) no serious multiple organ dysfunction of the heart, lungs, kidneys, and brain; and (5) all studies were performed by the same professional group.

The exclusion criteria were as follows: (1) patients who underwent pancreaticojejunostomy using the invaginated method; (2) patients with poor compliance and difficulty in strictly following the doctor’s advice; (3) patients with severe chronic diseases and an inability to tolerate surgery; (4) pancreatic tumors invading important blood vessels or distant metastasis; and (5) patients who discontinued treatment after surgery or did not regularly follow-up, resulting in incomplete perioperative data.

This retrospective study was conducted in accordance with the Helsinki Declaration and International Ethical Guidelines for Biomedical Research Involving Humans. The study was approved by the Medical Ethics Committee of the Second Affiliated Hospital of PLA Military Medical University (Supplementary Data Sheet 1). Informed consent was waived.

### Pancreaticojejunostomy

In traditional pancreaticojejunostomy, When shearing the pancreatic neck with surgical scissors, the reserved length of the pancreatic duct stump should exceed the pancreatic cross-section, which not only reduces the difficulty of anastomosis, but also facilitates the healing of the pancreatic duct to jejunal mucosa, and reduces the incidence of POPF. In addition, hemostasis of the bleeding in the pancreas section is required. When dealing with the stump of the pancreas, using an ultrasonic scalpel to dissociate the pancreas along the splenic vein with 1–2 cm.

### Modified Blumgart Anastomosis

Modified Blumgart anastomosis is an improvement on traditional pancreatic-intestinal anastomosis that is based on previous clinical practices. Modified Blumgart anastomosis for the treatment of pancreatic stump and Wirsung duct is in the same way as traditional pancreaticojejunostomy.

**Figure 1:**
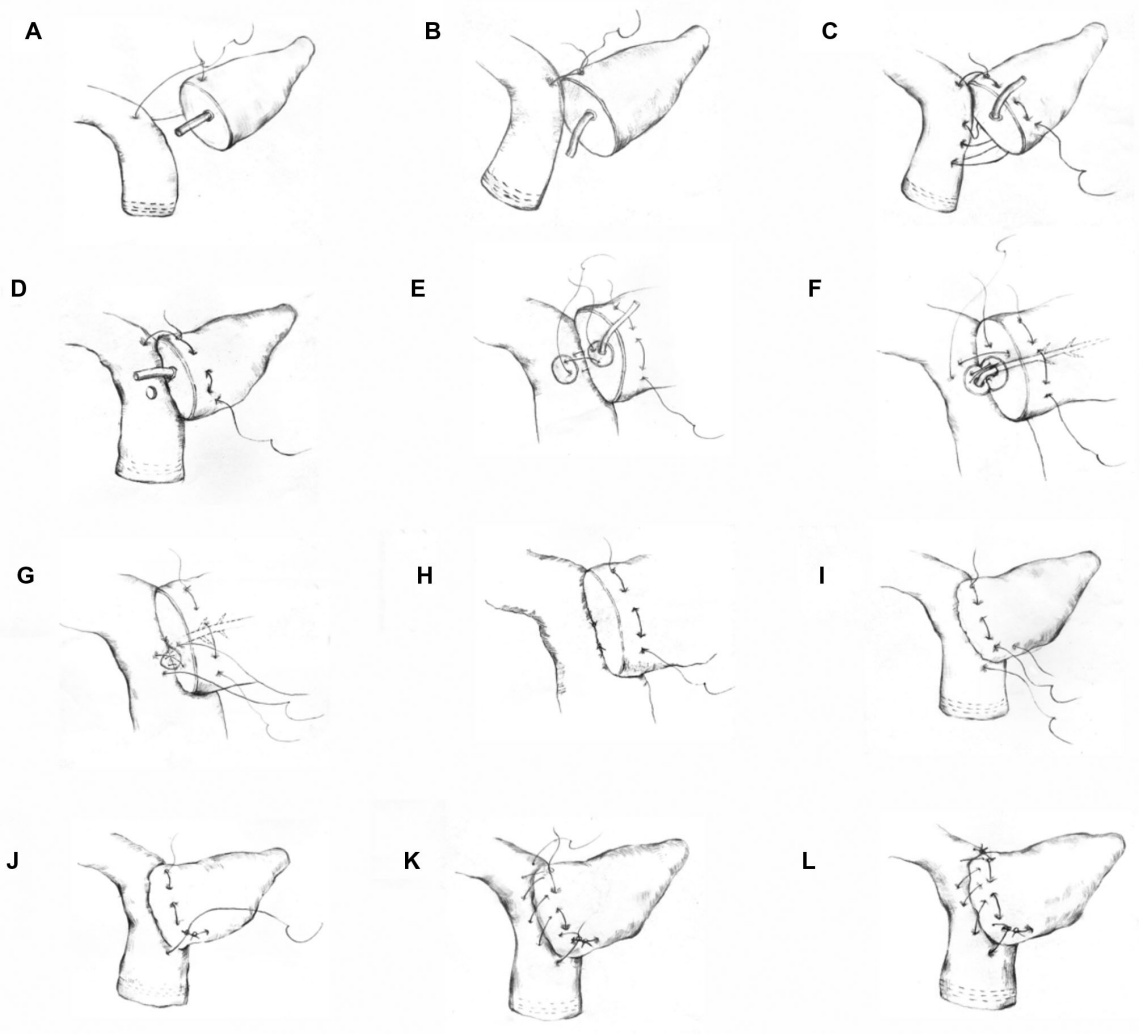
Schematic diagram of the modified Blumgart anastomosis. Step 1 (**A–D**): suturing of the posterior wall of the pancreas and jejunum. In steps (**A,B**) the superior border of the pancreas was penetrated. Then, the seromuscular layer of the jejunum was sutured and knotted. Step c shows the suturing and fixing of the superior border of the pancreas from the top to bottom through the entire pancreas and the suturing of the jejunum. Step 2 (**E–H**): the 8-shaped pancreaticojejunostomy anastomosis suture. Step (**D**) shows a small opening on the jejunum side made for the anastomosis. Step (**E**) shows the first 8-shaped stitch on the posterior wall of the pancreaticojejunostomy. Steps (**F,G**) show the 8-shaped sutures of the second and third stitches. Step (**I**) shows that the inferior border of the pancreas was penetrated through the complete layer. Then, the seromuscular layer of the jejunum was sutured and knotted with the unknotted suture in Step 1. Step 3 (**K,L**): the anterior wall of the pancreas was sutured with the seromuscular layer of the jejunum.

In the first step of this technique, a 3-0 Prolene suture (approximately 20 cm in length) was used to anastomose the posterior wall of the pancreas and intestine with the seromuscular layer of the jejunum. First, the stitch penetrated the superior border of the pancreas. Then, the seromuscular layer of the jejunum was sutured and a knot was tied (the tail was not cut). Suturing continued from top to bottom (the first step was to suture through the full thickness of the pancreas), and the last stitch was not knotted after the stitch was released (Figures 1A–D) (Supplementary Video 2). In the second step, according to the size of the pancreatic duct, a No.-6 or No.-8 silicone tube was used (an epidural tube was used for a pancreatic duct <2 mm) as a supporting tube to insert into the pancreatic duct. An electrocoagulation hook was used to make a small opening at the corresponding position on the opposite side of the pancreatic duct to avoid a large opening in the jejunum. A 5-0 PDS-II suture was used to suture the pancreatic duct and jejunum with three stitches in an “8” shape (equivalent to suturing a stitch in an “8” shape every 120 degrees; four stitches in an “8” shape can be made for a pancreatic duct >5 mm in diameter). Attention was given to ensure that the pancreatic duct along with pancreatic tissue that was 3 mm wide was sutured, if needed (Figures 1E–H). In the third step, suturing the anterior wall was performed in the foot-to-head direction. The first stitch was tied tightly, and then the unknotted suture in the first step was tightened and knotted with the suture in the third step. Next, the suture was sewn from the bottom to top and tightened and tied after the last stitch was pulled out (Figures 1I–L) (Supplementary Video 1).

### Outcomes

The primary outcome was to explore the association between the modified Blumgart anastomosis technique and operative time. The secondary outcome was to compare the incidence of complications between different surgical methods.

### Data Collection and Definitions

After the operation, one abdominal drainage tube was placed above the pancreatic duct-jejunal anastomosis and below the bile duct-jejunal anastomosis, respectively. The color and volume of the drainage fluid from the drainage tube were recorded every day after the operation, and drainage fluid amylase was checked on the 3rd and 5th postoperative day. If the amount of drainage amylase is less than 3 times the normal value and there is no ascites in the abdominal ultrasonography, the abdominal drainage tube will be removed. Routine gastrointestinal decompression and blood glucose monitoring were performed after the operation. When the patient felt hungry, the gastric tube was removed, water was given, and the patient was gradually transitioned to a normal diet according to the recovery of gastrointestinal function. All patients were given treatment including preventability anti-infection, parenteral nutrition, PPI, somatostatin injection et al.

The general data (age, sex, pathological diagnosis of the disease, body mass index, etc.), intraoperative factors (operation time, pancreaticojejunostomy time, and intraoperative blood loss), and postoperative results (gastrointestinal function recovery, hospitalization time, complications, and mortality) of all patients were collected and analyzed.

Common complications after laparoscopic PD include POPF, delayed gastric emptying, anastomotic hemorrhage, abdominal infection, bile leakage, venous thrombosis (portal, mesenteric, and splenic veins), chylous leakage, ascites, stress ulcer, and incision complications (11–13). The definition of pancreatic fistula according to the International Study Group on Pancreatic Fistula (12) is as follows: the amylase content in the peritoneal drainage fluid is more than three times that in serum on or after the third day following surgery (13). Delayed gastric emptying was defined as gastric stasis requiring nasogastric intubation for more than 7 days or reinsertion of a nasogastric tube after the failure of postoperative feeding. Delayed gastric emptying was diagnosed if one of the following conditions occurred after upper gastrointestinal radiography or gastroscopy, excluding mechanical factors such as anastomotic obstruction: (1) the gastric tube needed to be indwelled for >3 days after surgery; (2) after extubation, the gastric tube needed to be placed again due to vomiting and other reasons; and (3) the patient was unable to eat solid food until 7 days after surgery. For surgical site infection, the diagnostic criteria followed the definition by the National Nosocomial Infection Surveillance System of the US Centers for Disease Control and Prevention (14). The classification of recent postoperative complications followed the Clavien–Dindo classification system of 2004 (15).

### Statistical Analysis

SPSS 26.0 (SPSS Inc., IBM, Armonk, NY, USA) statistical software was used for data analysis in this study. The data are expressed as the mean ± standard deviation. The independent sample *t* test was used for comparisons between the two groups. The counting data were expressed by the incidence rate, and comparisons between the two groups were performed using the *χ*^2^ test. Multiple linear regression was used to analyze the factors influencing the operation time. A *P* value of <0.05 was considered to be statistically significant.

## Results

A total of 282 patients were enrolled in this study. The patients were divided into two groups according to the type of pancreaticojejunostomy. There were 177 patients in the traditional surgery group and 105 patients in the modified surgery group. The baseline characteristics of the enrolled patients are listed in Table 1. There were no statistically significant differences between the two groups in terms of sex, age, body mass index, American Society of Anesthesiology classification, or disease (*P* > 0.05).

**Table 1:** Patient characteristics and intraoperative factors.

Characteristics	Traditional group (*n *= 177)	Modified group (*n *= 105)	*P*
Gender (male/female)	105/72	62/43	0.964
Age (years)	58.85 ± 10.07	58.55 ± 11.7	0.823
BMI (kg/m^2^)*	23.23 ± 1.96	22.89 ± 3.89	0.411
Pancreatic duct diameter (mm)	3.43 ± 1.08	3.44 ± 2.01	<0.001
Operation time unit (min)	353.56 ± 50.27	323.8 ± 53.57	<0.001
Pancreaticojejunostomy time unit (min)	32.37 ± 3.47	21.41 ± 3.03	<0.001
Intraoperative blood loss (mL)	182.37 ± 78.31	185.43 ± 73.09	0.746
conversion to open surgery	12 (6.8%)	2(1.9%)	0.068
American Society of Anesthesiology classification			0.467
I	61 (34.5%)	43 (41%)	
II	101 (57%)	52 (49.5%)	
III	15 (8.5%)	10 (9.5%)	
Disease			0.324
Pancreatic cancer	43 (24.3%)	30 (28.6%)	
Cholangiocarcinoma	25 (14.1%)	14 (13.3%)	
Duodenal carcinoma	20 (11.3%)	14 (13.3%)	
Periampullary carcinoma	17 (9.6%)	18 (17.2%)	
Pancreatic neuroendocrine tumor	17 (9.6%)	6 (5.7%)	
Intraductal papillary mucinous neoplasm	20 (11.3%)	8 (7.6%)	
Duodenal stromal tumor	15 (8.5%)	4 (3.8%)	
Papillary adenoma of the duodenum	9 (5.1%)	4 (3.8%)	
Cystadenoma of the pancreas	9 (5.1%)	4 (3.8%)	
Pancreatic duct stones with chronic pancreatitis	2 (1.1%)	3 (2.9%)	

*P < 0.05 is considered statistically significant. Data are expressed as mean ± SD.*

The intraoperative factors of the two groups are listed in Table 1. During the operation, the amount of blood loss and conversion to open surgery in the traditional surgery and modified surgery groups were 182.37 ± 78.31 mL vs. 185.43 ± 73.09 mL and 12 (6.8%) vs. 2 (1.9%), respectively. There were no statistically significant differences between the two groups (*P* > 0.05). The diameter of the pancreatic duct and operation time in the traditional and modified groups were 3.43 ± 1.08 mm vs. 3.44 ± 2.01 mm and 353.56 ± 50.27 min vs. 323.8 ± 53.57 min, respectively. The pancreaticojejunostomy time in the traditional and modified groups was 32.37 ± 3.47 min vs. 21.41 ± 3.03 min, respectively. There were statistically significant differences between the two groups (*P* < 0.001).

The postoperative complications are listed in Table 2. When comparing the incidence of postoperative complications between the two groups, no statistically significant differences were observed in the Clavien–Dindo classification, reoperation, bleeding, bile leakage, chylous leakage, pulmonary infection, abdominal infection, delayed gastric emptying, or ascites. However, the incidence of POPF in the traditional surgery and modified groups was 45 cases (25.4%) and 11 cases (10.5%), respectively. These cases included 10 cases in the traditional surgery group and 4 case in the modified group with POPF grades B. There were 4 cases of grade C POPF in the traditional surgery group and none in the modified group, and this was significantly different between the two groups (*P* = 0.003). No deaths were recorded in either group. All patients were followed up for 3–9 months, and there was no readmission due to complications. The results of the linear regression analysis showed that the influence of the surgical method on operation time was statistically significant (*P* < 0.001) and that the surgical method was associated with operative time. The operation time of the modified group was significantly shorter than that of the reference group (95% CI, −73.074 to −23.941, β: −0.438, *P* < 0.001; Table 3).

**Table 2:** Incidence of postoperative complications.

Postoperative complications	Traditional group (*n *= 177)	Modified group (*n *= 105)	t/X^2^	*P*
Clavien–Dindo Classification			13.092	0.004
Grade I	41 (23.2%)	12 (11.4%)		
Grade II	34 (19.2%)	11 (10.5%)		
Grade III	10 (5.6%)	5 (4.8%)		
Grade IV–V	0 (0%)	0 (0%)		
Surgical re-intervention	6 (3.4%)	2 (1.9%)	0.527	0.468
Bleeding	21 (11.9%)	5 (4.8%)	3.972	0.046
Gastrointestinal bleeding	14 (7.9%)	4 (3.8%)		
Intraperitoneal hemorrhage	7 (4%)	1 (0.95%)		
Postoperative pancreatic fistula	45 (25.4%)	11 (10.5%)	22.183	<0.001
Biochemical fistula	31 (17.5%)	7 (6.7%)		
Grade B	10 (5.6%)	4 (3.8%)		
Grade C	4 (2.3%)	0 (0%)		
Bile leakage	6 (3.4%)	2 (1.9%)	0.527	0.468
Chylous leakage	2 (1.1%)	1 (0.95%)	0.020	0.888
Pulmonary infection	6 (3.4%)	2 (1.9%)	0.527	0.468
Abdominal infection	5 (2.8%)	2 (1.9%)	0.230	0.631
Delayed gastric emptying	12 (6.8%)	4 (3.8%)	1.086	0.297
Ascites	2 (1.1%)	1 (0.95%)	0.020	0.888

*P < 0.05 is considered statistically significant.*

**Table 3:** Independent factors affecting operation time.

Variables	β	SE	Standardization β	95% CI	*P* values
Type of surgery					
Traditional	Reference				
Modified	−48.507	12.476	−0.438	−73.074 to −23.941	<0.001
BMI	0.069	1.106	0.004	−2.109 to −2.247	0.951
Age	0.314	0.292	0.063	−0.26 to −0.889	0.283
Intraoperative blood loss	0.025	0.041	0.036	−0.056 to −0.106	0.541
Pancreaticojejunostomy time	−1.784	0.958	−0.208	−3.67 to −0.103	0.064
Pancreatic duct diameter	0.158	2.151	0.005	−4.076 to −4.393	0.941
Disease					
Periampullary carcinoma	Reference				
Pancreatic cancer	−27.344	14.786	−0.225	−56.325 to −1.637	0.062
Intraductal papillary mucinous neoplasm	−22.554	13.476	−0.127	−49.088 to −3.981	0.095
Duodenal stromal tumor	−19.036	14.866	−0.090	−48.308 to −10.235	0.201
Cystadenoma of the pancreas	−13.544	16.846	−0.053	−46.715 to −19.627	0.422
Papillary adenoma of the duodenum	−11.304	16.969	−0.045	−44.717 to –22.109	0.506
Cholangiocarcinoma	−18.425	12.175	−0.119	−42.399 to −5.548	0.131
Pancreatic neuroendocrine tumor	1.605	14.385	0.008	−26.72 to −29.93	0.911
Duodenal carcinoma	−31.222	15.939	−0.191	−62.462 to −0.018	0.093
Pancreatic duct stones with chronic pancreatitis	−38.155	24.983	−0.095	−87.349 to −11.038	0.128
American Society of Anesthesiology classification					
I	Reference				
II	−0.173	6.703	−0.002	−13.371 to −13.024	0.979
III	−1.586	11.951	−0.008	−25.118 to −21.947	0.895

*P < 0.05 is considered statistically significant.*

## Discussion

In the present study, the surgical method was associated with the operative time. Our modified technique reduces the operative time and potentially reduces life-threatening surgical complications.

At present, there are many methods for the reconstruction of the residual pancreas and digestive tract after PD. The two most common methods are pancreaticojejunostomy and pancreatogastrostomy (16). Most randomized controlled trials show that there is no remarkable difference in the incidence of postoperative POPF between pancreaticojejunostomy and pancreatogastrostomy (17, 18). However, some studies have revealed that pancreatogastrostomy is markedly better than pancreaticojejunostomy (19, 20). Although the best anastomosis procedure for digestive tract reconstruction is controversial, pancreaticojejunostomy remains the preferred choice of most surgeons (21–24). The commonly used methods for pancreaticojejunostomy include invaginated and duct-to-mucosa pancreaticojejunostomy, and improvements in these two techniques have emerged in quick succession. Invaginated pancreaticojejunostomy involves the complete invagination of the cut end of the pancreas into the jejunum cavity and suturing of the two layers. The advantages of this procedure are that the operation is simple and suitable for all patients, and the pancreatic fluid in the main pancreatic duct and pancreatic stump can enter the intestinal canal. However, using this anastomosis method leaves the pancreatic section completely exposed in the intestinal cavity, which may result in fatal massive hemorrhage. Moreover, invagination is not an easy procedure to perform in cases of pancreatic hypertrophy. Once a POPF occurs, it often causes anastomotic dehiscence, which can lead to serious abdominal infection (25).

Pancreatic duct-to-mucosa anastomosis allows for rapid and better healing without exposure to pancreatic fluid. Therefore, the anastomotic stoma heals quickly. Furthermore, the pancreatic section is surrounded by the seromuscular layer during pancreaticojejunostomy, which reduces the risk of bleeding and maintains long-term patency of the pancreatic duct. It ensures straightforward entry of pancreatic juice into the intestinal cavity, maintenance of pancreatic exocrine function, and improvement in the postoperative quality of life of patients. Therefore, this anastomosis method is recommended by pancreatic surgery experts worldwide (21–24). Despite these advantages, duct-to-mucosa pancreaticojejunostomy has the following limitation (7): dead space may be left between the surface of the pancreatic section and jejunum wall, which may cause fluid retention. It is difficult to perform pancreatic duct-to–mucosal anastomosis in cases with a small and thin pancreatic duct. In addition, Blumgart (25) and Hirono (26) reported that the rate of complications with Blumgart anastomosis was remarkably lower than that with invaginated and duct-to-mucosa and end-to-side pancreaticojejunostomy. Blumgart anastomosis is a simple and safe pancreaticojejunostomy procedure that reduces the incidence of POPF grades B and C after surgery. The greatest advantage of this technique is attributed to the reliable adhesion between the dorsal wall of the inferior border of the pancreas and the seromuscular layer of the jejunum; however, surgery using a laparoscope is challenging.

The criteria for an “ideal” pancreaticojejunostomy include a low incidence of POPF, a low rate of complications, a wide application range, and an easy to learn method. Based on previous clinical practice using pancreaticojejunostomy, we propose a new anastomosis technique with a simple operation process and low incidence of POPF, making it convenient for popularization in hospitals at all levels. This new pancreaticojejunostomy technique involves double-layer suturing. The outer layer is an improved suture based on Blumgart anastomosis (currently considered to have the lowest incidence of POPF), which improves the simplicity while retaining its effectiveness. This improvement involves two parts.

In the first part, a 3-0 Prolene suture is used to continuously penetrate the entire thickness of the pancreas, followed by suturing the seromuscular layer of the jejunum. This technique has several advantages. It reduces the suturing steps, lowers the difficulty of surgical suturing, and shortens the operation time. Also, the same anastomotic sealing performance and low incidence of POPF are retained because the full thickness of the pancreas is penetrated. Furthermore, compared to an intermittent mattress suture, it preserves the blood supply of the anastomosis and has better advantages than the traditional Blumgart anastomosis.

In the second part, for anastomosis of the pancreatic duct and jejunum mucosa-to-mucosa, three 8-shaped stitches to the pancreatic duct mucosa-to-jejunal mucosa are used for equal division of the anastomotic stoma (one circle of the anastomotic stoma is 360 degrees). This is equivalent to suturing one 8-shaped stitch every 120 degrees (four stitches in the shape of an 8 can be made for a pancreatic duct >5 mm in diameter). Compared to the traditional intermittent suture method of mucosa-to-mucosa, this improvement has several advantages. The tension is more uniform, which reduces blood loss due to cutting of the tissue and providing better sealing of the anastomosis. In traditional intermittent suturing, there are differences in the knotting strength of each stitch. Thus, it is not easy to maintain uniform tension at each knot, which may lead to increased cutting of some tissues due to knotting. This may result in physical microleakage and pancreatic leakage after further expansion. Some knots are not tightly secured, resulting in physical leakage. Knotting after the 8-shaped anastomosis suture will disperse and transfer the tension to the two joints, as per mechanical principles. Thus, it reduces cutting and damage to the tissue to ensure the sealing performance of the anastomosis. An additional advantage is that compared to Blumgart intermittent anastomosis, the surgical difficulty and steps in this process are greatly reduced, especially with the use of a laparoscope. Intermittent mucosa-to-mucosa suturing is complicated and has high technical requirements. However, this method has more advantages with the 8-shaped sutures than the intermittent sutures by reducing the knotting time by half and avoiding suture clutter. In particular, laparoscopic anastomosis is simple and reduces the anastomosis time by 20% compared with laparotomy and 34% compared with laparoscopic anastomosis.

This anastomosis method is new and has been designed on the basis of a mechanical analysis and the biological characteristics of mechanical anastomosis. Moreover, its improvements and enhancements are based on its predecessors, having the characteristics of less physical leakage and better biological healing. In addition, it reduces the difficulty involved in pancreaticojejunostomy, especially laparoscopic pancreaticojejunostomy, and markedly shortens the operation time. Therefore, it has good popularization value and technical predictability. This method of anastomosis mitigates the incidence of bleeding and POPF of the branching small pancreatic duct. Even if POPF occurs, the maximum range of the leakage is less than 1/3. Thus, it can be conservatively treated and rapidly healed. We used a modified Blumgart anastomosis in our study and observed no complications, such as bleeding or death. The incidence of POPF was reduced, the surgical difficulty was decreased, and the operation time was decreased from 25–40 min to 15–30 min.

We performed laparoscopic PD with modified Blumgart anastomosis in 105 patients. In our practice, laparoscopic PD can be performed in pancreatic ducts with diameters <2 mm using a modified Blumgart anastomosis. The key to a successful procedure is to determine the position of the pancreatic duct. When the pancreatic duct is located after dissecting the pancreatic neck, scissors are used to cut the pancreatic duct. At this stage, care is taken to retain as much of the pancreatic duct as possible to facilitate tissue healing. Compared to intermittent anastomosis, continuous anastomosis with pancreaticojejunostomy has the following benefits: continuous suturing is a simple, fast, and safe technique that can reduce the incidence of POPF, and the distribution of tension is uniform with this technique. Continuous suturing avoids intermittent multiple suturing of the pancreas, reduces damage to the pancreatic parenchyma, decreases the number of pancreatic pinholes, and lowers the risk of pancreatic fluid leakage from pinholes. Moreover, if one stitch is sutured and one knot is tied during intermittent suturing, a narrow gap is created between the jejunum and pancreas, which makes it an unsuitable position for the next stitch.

Despite our promising findings, our study has some limitations. This is a retrospective study. Therefore, it lacks foresight and randomness and may have biases. Our operations were performed by doctors in the same surgical group. It is impossible to ensure that other doctors with the same experience perform the same surgical techniques. The surgeons improved their technique through repetition after performing more than 200 operations.

## Conclusion

This modified Blumgart anastomosis is a reliable, fast, simple, and easy-to-learn procedure with a low risk of POPF, and it was found to be associated with reduced operation time.

## Data Availability

The original contributions presented in the study are included in the article/Supplementary Material, further inquiries can be directed to the corresponding author/s.
