# Supplementary material for: Association of a Modified Blumgart Anastomosis With the Incidence of Pancreatic Fistula and Operation Time After Laparoscopic Pancreatoduodenectomy: A Cohort Study
Source: Front Surg. 2022 Jun 27;9:931109. doi: 10.3389/fsurg.2022.931109 (PMC9271827; doi:10.3389/fsurg.2022.931109)
Supplement: Supplementary file 1 [file Data_Sheet_1_v1.pdf]

文件编号：AF/SC-08/1.0

审查批件

<table>
<tr><td>伦理审查编号</td><td colspan="3">2018-研第 045-01</td></tr>
<tr><td>项目名称</td><td colspan="3">3D 腹腔镜和开腹胰十二指肠切除术的随机化对照临床试验</td></tr>
<tr><td>项目来源</td><td colspan="3">国家自然科学基金
组长单位：同济医院</td></tr>
<tr><td>临床研究机构</td><td>肝胆外科</td><td>主要研究者</td><td>李靖</td></tr>
<tr><td>审查类别</td><td>初始审查</td><td>审查方式</td><td>会议审查</td></tr>
<tr><td>审查时间</td><td>2018-9-26</td><td>审查地址</td><td>药学部 3 楼药理基地会议室</td></tr>
<tr><td>审查委员</td><td colspan="3">详见“会议签到表”</td></tr>
<tr><td>送审文件</td><td colspan="3">1. 初始审查申请
2. 研究方案（版本号 V1.0，版本日期 2018 年 3 月 20 日）
3. 知情同意书（版本号 V1.0，版本日期 2018 年 3 月 20 日）
4. 病例报告表（版本号 V1.0，版本日期 2018 年 3 月 20 日）
5. 研究经济利益声明
6. 主要研究者简历和 GCP 证书
7. 其他研究人员的简历、GCP 证书
8. 组长单位伦理批件
9. 关于国家自然科学基金资助项目批准及有关事项的通知</td></tr>
<tr><td>审查决定</td><td colspan="3">同意。跟踪审查频率：12 个月，截止日期为 2019 年 9 月 25 日</td></tr>
<tr><td>注意事项</td><td colspan="3">1、请遵循 GCP 原则、赫尔辛基宣言和伦理委员会批准的方案开展临床研究，保护受试者的健康与权益。
2、研究开始前，请按有关职能部门或机关的要求完成临床研究的注册或立项手续。
3、研究过程中若需变更主要研究者或对方案、知情同意书、招募材料等重要材料需进行任何修改，请提交修正案审查申请。
4、本中心若发生严重不良事件，请及时提交严重不良事件报告。
5、请按照跟踪审查频率，在截止日期前 1 个月提交研究进展报告；我</td></tr>
</table>

	中心作为多中心研究项目组长单位的，还应提交各中心研究进展的汇总报告；当出现任何可能显著影响试验进行或增加受试者风险的情况时，请及时向伦理委员会提交书面报告。<br>6、研究过程中若出现纳入了不符合纳入标准或符合排除标准的受试者、符合中止试验规定而未让受试者退出研究、给予错误治疗或剂量、给予方案禁止的合并用药等没有遵从方案开展研究的情况或可能对受试者的权益/健康以及研究的科学性造成不良影响等违背 GCP 原则的情况，请提交违背方案报告。<br>7、暂停或提前终止临床研究，请及时提交暂停/终止研究报告。<br>8、完成临床研究，请提交研究完成报告。<br>9、本临床试验应在批准之日起 1 年内实施，逾期未实施的，本批件自行废止。
声明	本伦理委员会严格按照中国 GCP、ICH-GCP 和有关法规对送审的中文纸质材料进行评审（对送审的光盘及外文纸质材料不作评审仅备案），其审查工作过程不受伦理委员会以外的任何组织及个人的影响。
伦理委员会名称	中国人民解放军陆军军医大学第二附属医院医学伦理委员会（盖章）
联系方式	伦理委员会办公室联系人：邓璠　023-68755422
地址	陆军军医大学第二附属医院药学部三楼伦理委员会办公室

主任委员签字	任谦	日期	2018 年 10 月 16 日
